# Nanoparticle core stability and surface functionalization drive the mTOR signaling pathway in hepatocellular cell lines

**DOI:** 10.1038/s41598-017-16447-6

**Published:** 2017-11-22

**Authors:** Mariia Lunova, Andrey Prokhorov, Milan Jirsa, Martin Hof, Agnieszka Olżyńska, Piotr Jurkiewicz, Šárka Kubinová, Oleg Lunov, Alexandr Dejneka

**Affiliations:** 1 Institute for Clinical & Experimental Medicine (IKEM), Prague, Czech Republic; 2 Institute of Physics of the Czech Academy of Sciences, Prague, Czech Republic; 3 J. Heyrovský Institute of Physical Chemistry AS CR, v.v.i., Dolejškova 2155/3, 182 23 Prague 8, Czech Republic; 4 Institute of Experimental Medicine, the Czech Academy of Sciences, Prague, Czech Republic

## Abstract

Specifically designed and functionalized nanoparticles hold great promise for biomedical applications. Yet, the applicability of nanoparticles is critically predetermined by their surface functionalization and biodegradability. Here we demonstrate that amino-functionalized polystyrene nanoparticles (PS-NH_2_), but not amino- or hydroxyl-functionalized silica particles, trigger cell death in hepatocellular carcinoma Huh7 cells. Importantly, biodegradability of nanoparticles plays a crucial role in regulation of essential cellular processes. Thus, biodegradable silica nanoparticles having the same shape, size and surface functionalization showed opposite cellular effects in comparison with similar polystyrene nanoparticles. At the molecular level, PS-NH_2_ obstruct and amino-functionalized silica nanoparticles (Si-NH_2_) activate the mTOR signalling in Huh7 and HepG2 cells. PS-NH_2_ induced time-dependent lysosomal destabilization associated with damage of the mitochondrial membrane. Solely in PS-NH_2_-treated cells, permeabilization of lysosomes preceded cell death. Contrary, Si-NH_2_ nanoparticles enhanced proliferation of HuH7 and HepG2 cells. Our findings demonstrate complex cellular responses to functionalized nanoparticles and suggest that nanoparticles can be used to control activation of mTOR signaling with subsequent influence on proliferation and viability of HuH7 cells. The data provide fundamental knowledge which could help in developing safe and efficient nano-therapeutics.

## Introduction

The fast nanotechnology advancements in recent years resulted in the development of numerous nanomaterials, which often possess complex structures and surface functionalization^1–3^. Biomedical applications (for example, imaging, diagnosis, drug delivery, etc.) of functionalized nanoparticles (NPs) are steadily increasing^1–4^. Due to small particle size and their large surface area, NPs possess chemical and physical properties that cannot be achieved by the corresponding bulk materials^5^.

Despite enormous progress in the development of novel therapies, conventional cancer therapies still retain the intrinsic limitations that prompted the development and application of various nanotechnologies for more effective and safer cancer treatment^6,7^. Indeed, several therapeutic NP platforms are already under evaluation and great promise in clinical development is expected with definitive results to be available in the near future^8^. However, despite intense investigations and progress in the field of cancer nanomedicine, it has been criticized that translation of the results from small animal models to successful clinical applications is very limited^9^.

It is generally believed that tumor tissues accumulate most of therapeutic systematically administered NPs through the enhanced permeability and retention (EPR) effect^8,10,11^. However, multiple biological factors in the systemic delivery of NPs can dramatically influence the efficiency of the delivery and therapeutic effects. NP–protein interaction in blood, NP uptake by macrophages, extravasation into and interaction with the perivascular tumor microenvironment, tumor tissue penetration and tumor cell internalization represent examples of these factors^8^. Thus, NP biological activity is critically determined by their surface functionalization, which procures contact with the surrounding media. In order to predict the fate of injected NPs, it is important to understand the interactions occurring at the interface between NPs and biological components.

After endocytosis, most nanomaterials will eventually accumulate in acidic vesicular organelles, such as endosomes and lysosomes^2,12,13^. The hydrolytic enzymes in these organelles represent a hostile environment for endocytosed nanomaterials causing their degradation. Importantly, malignant and invasive cancer cells strongly depend on properly functioning acidic organelles. In transformed cells, lysosomal stability, trafficking and composition are frequently altered. Cancer cells display lysosome hypertrophy because of increased lysosomal hydrolases secretion which is important for tumor progression. Hypertrophy renders lysosomes fragile by increasing lysosomal membrane permeabilization (LMP)^14,15^. Therefore, targeting lysosomes to trigger lysosomal leakage may be utilized for cancer therapy. Such an approach could be associated with fewer side effects and higher therapeutic efficacy due to evasion of common resistance mechanisms^16^. Moreover, it has been shown that cationic amphiphilic drugs (CADs) selectively kill cancer cells via LMP^17^. Additionally, we and others have shown previously that amino-functionalized NPs can induce lysosomal swelling and result into cancer cell death^12,13,18,19^.

A key kinase controlling cell growth and proliferation under favorable environmental conditions is the mammalian target of rapamycin (mTOR). Membranes limiting acidic lysosomal compartments are important for the activation of mTOR^20,21^. mTOR as well as some of the targets of the mTOR kinase signaling are overexpressed or mutated in cancer, and it is regarded as a promising target for anticancer treatment^20,21^. It is worth noting here, that mTOR inhibitors display favorable pharmacological profiles and are well tolerated comparing to conventional anticancer therapy^22^.

Recent research demonstrated that various NPs modulate the activation of mTOR and even may result into cell cycle arrest in leukemia cells^19,23–25^. More specifically, amino-functionalized NPs have been shown to inhibit mTOR activity and proliferation in three leukemia cell lines^19^. However, current knowledge of the physiological, pathophysiological effects of NPs on liver cells remain unclear. mTOR is frequently up-regulated in cancer including hepatocellular carcinoma (HCC) and its upregulation is associated with bad prognosis, poor tumor differentiation and earlier recurrence^26^. Therefore, in the present study we investigated NPs of different core composition functionalized either with amino or hydroxyl groups as a platform for targeting lysosomes and mTOR signaling in liver derived cell lines Huh7 and HepG2.

In order to adress this question, we used polystyrene (PS) and silica NPs as model particles for our experiments. PS NPs possess many advantages, including straightforward synthesis in a wide range of sizes, relatively low costs, easy separation, and easy surface modification^27^. Furthermore, PS NPs were recognized as biologically inert, almost non-biodegradable and fully biocompatible^28–31^. These properties make PS NPs a perfect tool for studying biomedical consequences of various surface modifications^27^. Contrary to PS, silica NPs represent an excellent model of biodegradable biocompatible type of NPs suitable as a potential drug delivery system^32–34^. Therefore, we used those properties to study the effects of surface functionality on the NP cell interaction. A detailed understanding of the impact of the surface functionalization is a prerequisite for the rational design of nanomaterials targeting distinct cell types for diagnostic or therapeutic purposes. Additionally, we investigated the role of a protein corona in these effects, taking into account that the protein corona can give rise to undesirable adverse effects^35^.

## Results

### Characterization of BSA and RNase interaction with functionalized nanoparticles

**Figure 1:**
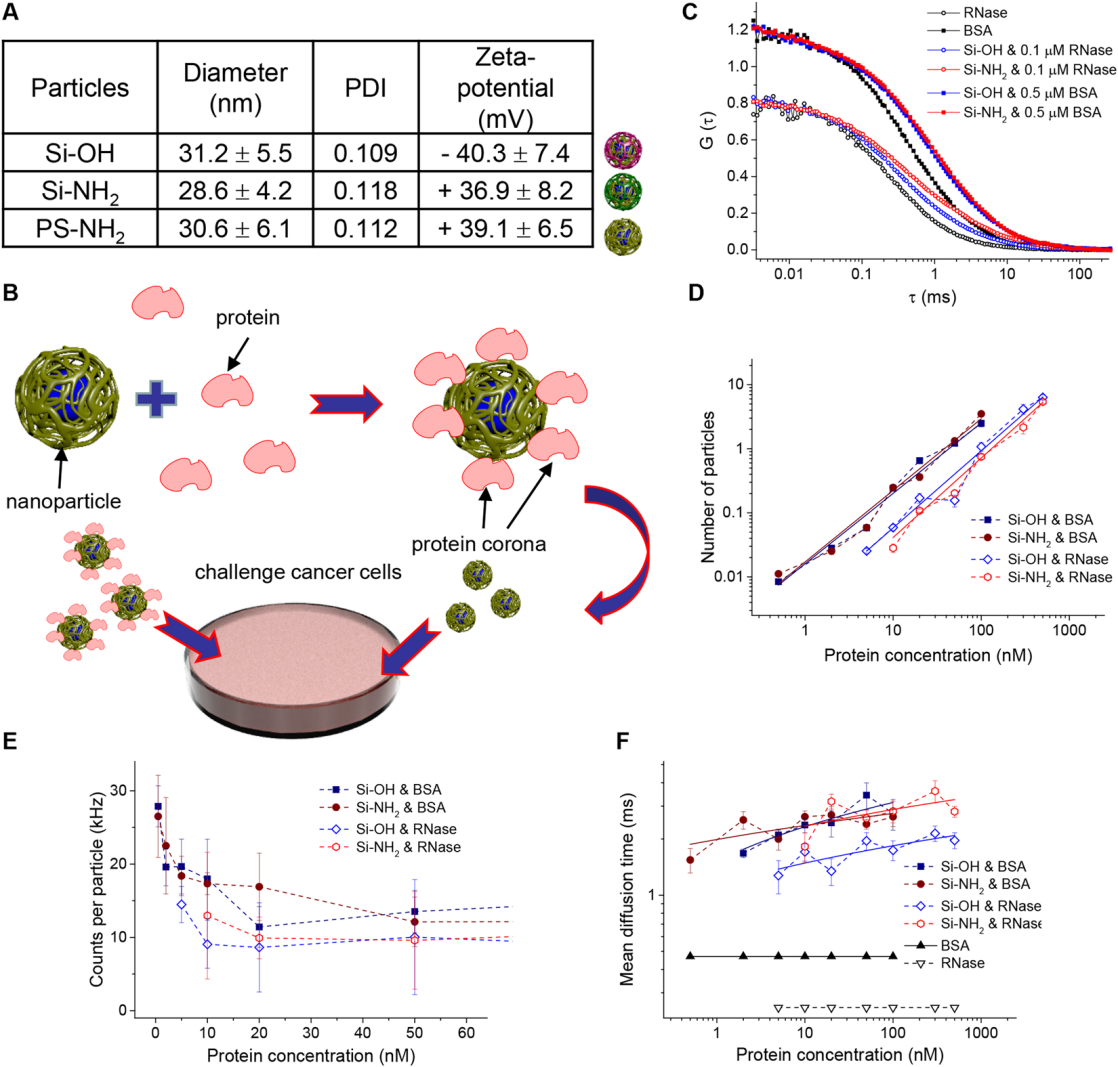
Physicochemical characterization of the chemically distinct nanoparticles. (**A**) Characterization of hydroxyl-, amino-functionalized silica and amino-functionalized PS NPs. Physical parameters of NPs were characterized by dynamic light scattering (DLS) using a Zetasizer Nano. PDI, polydispersity index. (**B**) Scheme of the experimental setup. Protein adsorption on silica NPs characterized using fluorescence correlation spectroscopy. Positively (Si-NH_2_) and negatively (Si-OH) charged silica NPs were titrated with fluorescently labeled BSA and RNase. (**C**) Exemplary autocorrelation curves normalized to 0.8 or 1.2. (**D**) Number of particles, (**E**) their brightness, and (**F**) mean diffusion time. Curves in logarithmic plots (**D**,**F**) were fitted with straight lines according to Freundlich adsorption model. Error bars show SEM for n = 3.

**Figure 2:**
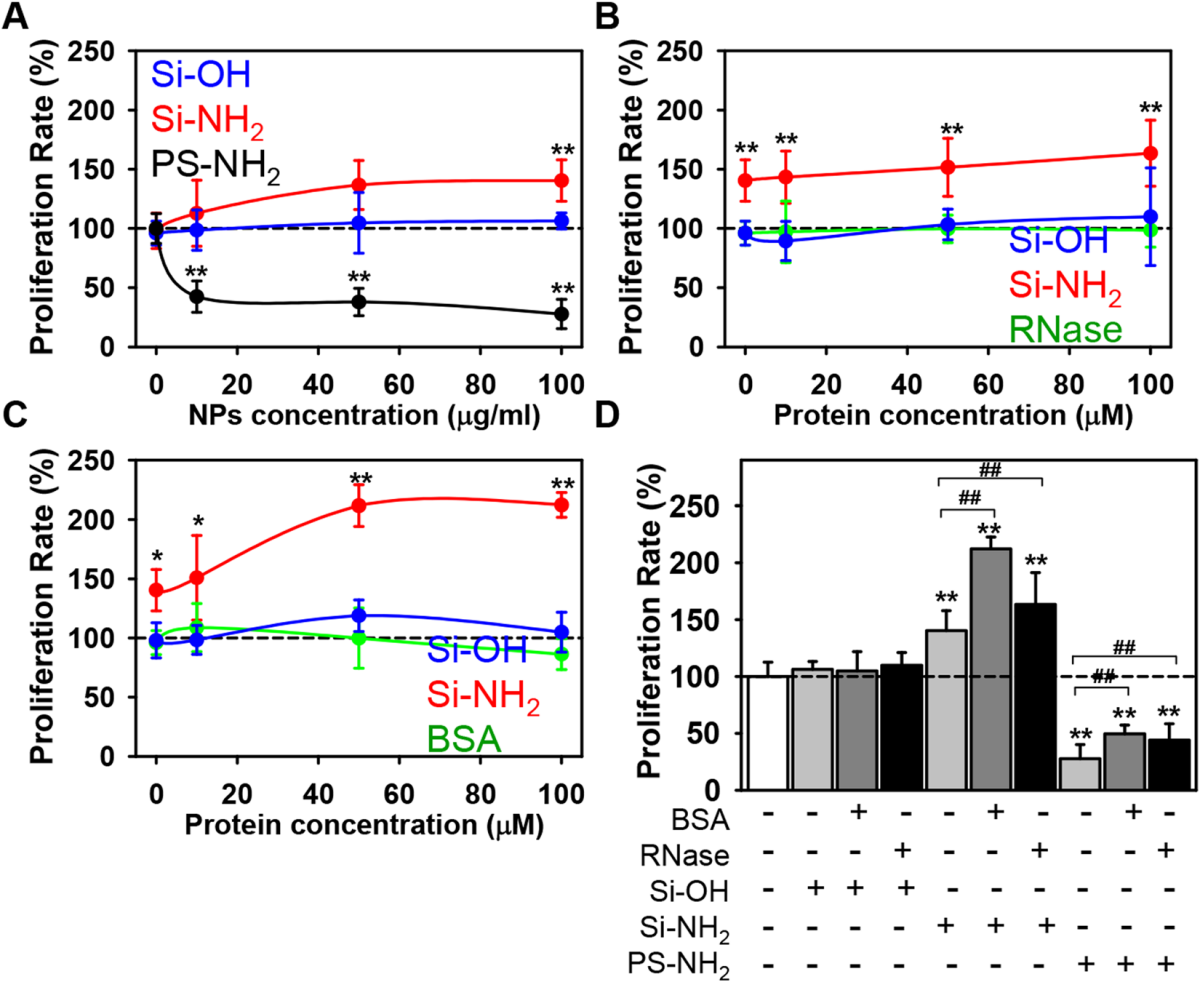
Inhibition of proliferation in Huh7 cell line by PS-NH_2_ nanoparticles. (**A**) Cells were cultured in medium for 24 h in the presence or absence of hydroxyl- (Si-OH), amino-functionalized (Si-NH_2_) silica, or amino-functionalized (PS-NH_2_) PS NPs. Cell viability was assessed by the WST-1 assay. The data were normalized to control values (no particle exposure) and expressed as mean ± SEM, n = 3 each. (**B**) Analysis of cytotoxicity in Huh7 cultured with Si-OH, Si-NH_2_, or PS-NH_2_ NPs bearing RNase as hard protein corona. Cells were cultured in the presence or absence of Si-OH or Si-NH_2_ NPs (all 100 µg/ml) pre-incubated with increasing concentrations of RNase for 1 h. Cell viability was assessed by the WST-1 assay. The data were normalized to control values (no particle exposure) and expressed as mean ± SEM, n = 3 each. (**C**) Analysis of cytotoxicity in Huh7 cultured with Si-OH or Si-NH_2_, NPs bearing BSA as hard protein corona. Cells were cultured in the presence or absence of Si-OH or Si-NH_2_ NPs (all 100 µg/ml) pre-incubated with increasing concentrations of BSA for 1 h. Cell viability was assessed by the WST-1 assay. The data were normalized to control values (no particle exposure) and expressed as mean ± SEM, n = 3 each. (**D**) Comparison of proliferative activity of Huh7 cultured with Si-OH, Si-NH_2_, or PS-NH_2_ NPs bearing BSA or RNase (100 µM both) as hard protein corona or bare NPs. Cell were treated as in (**A**–**C**). The data were normalized to control values (no particle exposure) and expressed as mean ± SEM, n = 3 each.

Generally, when administrated intravenously NPs become coated with proteins and other biomolecules that form a so-called protein corona^36^. It is widely accepted that NPs are internalized into the cell and traffic along defined pathways such as the endo-lysosomal pathway. It has also been shown that specific proteins present in the original protein corona are retained on NPs until they accumulate in lysosomes^37^. Consequently, these retained proteins may play an important role in determining subsequent cellular processing^36,37^. Therefore, it is of great importance to determine how chemical surface modification of NPs and subsequent NP-protein interactions affect biological responses. In our study we utilized three types of NPs as a platform allowing to gain insight into biomedical relevant key determinants of surface functionalization of NPs. The physicochemical properties of the NPs were investigated by dynamic light scattering (summarized in Fig. 1A). All three types of particles had the same mean hydrodynamic diameter of about 30 nm. The particles were functionalized either with hydroxyl or amino groups as reflected by high negative and positive zeta potential, respectively (Fig. 1A). Similar physicochemical characteristics of the particles and the low polydispersity index (PDI) allowed us to analyze the role of the surface charge on the protein corona formation and subsequent pathophysiological responses of cancer cells (Fig. 2B). Previously, we and others showed that amino-functionalized polystyrene NPs (PS-NH_2_) may result into lysosomal rupture associated with apoptotic cell death^12,18,19,38,39^. Therefore, in this study we addressed a question whether other types of amino-functionalized NP may lead to the same functional consequences. We selected amino-functionalized silica NPs (Si-NH_2_) as an alternative to PS-NH_2_. Among inorganic-based materials, silica NPs have attracted much research attention for their potential application in nanomedicine, especially as suitable drug delivery system^32–34^. We did not utilize as an alternative for non-biodegradable NPs metallic inorganic particle (e.g. gold) due to the fact that in contrast to polystyrene gold NPs are not inert^40^. Additionally, gold or other metal NPs release ions from the NPs^41^. Indeed, intracellular gold ions are known to strongly inhibit the enzymatic activity of cells, leading to mitochondrial membrane depolarization and/or inactivation of mitochondrial enzymes^42^. Therefore, such NPs cannot represent a reliable model to study NP surface functionalization-related cellular effects.

As model proteins we selected serum albumin (a major soluble constituent of human blood plasma and thus a relevant target for studies of nanoparticle–protein interactions^36,43,44^) and ribonuclease A (a protein the serum levels of which are elevated in pancreatic carcinoma patients^45^). Our intention was to compare abundant protein in serum at normal conditions with the protein that is elevated in cancer pathology.

Dispersion of silicon dioxide NPs was titrated with fluorescently labeled proteins albumin (BSA) or ribonuclease A (RNase). Their adsorption on the NP surface was monitored using fluorescence correlation spectroscopy (FCS), which allowed determination of particle size and concentration^46^. To modify the electric charge of the NPs, they were functionalized either with either hydroxyl or amino groups (Fig. 1A). Examples of the obtained autocorrelation curves are shown in Fig. 1C. Their amplitudes were normalized for easier comparison. After mixing proteins with NPs the curves shifted to the right, which is a result of the slow-down of the diffusion of protein molecules upon binding to the NPs. While titrating the NPs with fluorescent proteins the mean number of the diffusion entities increased (Fig. 1D). These entities included protein-coated NPs, the number of which was constant, as well as free protein molecules, the number of which increased during the titration. While the number of particles gave us a control over the protein concentration, a better marker of the adsorption was a count per particle – the mean brightness of the diffusing particles (i.e. fluorescence intensity divided by the number of particles) (Fig. 1E). At low protein concentrations most of the protein molecules were adsorbed and diffused together with the NPs, therefore the mean particle brightness was relatively high. At higher protein concentrations the adsorption sites saturated and the fraction of free protein molecules increased. Since the brightness of a free protein was lower than the brightness of the NP with many adsorbed proteins, the mean particle brightness decreased. Above 20 nM of protein count per particle leveled out. The mean diffusion times of the free proteins (0.25 ms for RNase and 0.47 ms for BSA) depicted as triangles in Fig. 1F were measured in a separate experiments and fixed during the fitting of the data from the NP titration. The second diffusion time corresponded to the diffusion of the NPs with adsorbed proteins. It increased slightly with increasing protein concentration, which reflected slight tendency of the coated NPs to aggregate.

Taking together, these data indicate that saturation in protein corona formation was reached at physiological concentrations of both proteins in human blood plasma^36,43–45^. Formation of protein corona was very rapid in time reaching an equilibrium within one hour. These results provided a platform for protocol optimization for experiments with cell treatment.

### Amino-functionalized polystyrene NPs inhibit whereas amino-functionalized silica NPs enhance proliferation of hepatocellular carcinoma cells

In our previous works we analyzed the mechanisms of functionalized NP uptake by different cell lines as well as cytotoxic potential of amino-functionalized NPs^13,38^. Moreover, we and others have shown that positively charged NPs may passively target tumor cells even *in vivo* ^8,13,47^. Numerous studies have shown that functionalization of NPs with amino groups improves passive tumor targeting via EPR effect^19,48–51^. Therefore, based on these data in our experiments, we studied interaction of amino-functionalized NPs with cells as a tentative model for passive tumor targeting. PS and silica NPs served solely as model particles providing a convenient nanosized platform to study biomedical consequences of surface modifications and functionalization.

NPs surface-functionalized with amino groups can induce lysosomal swelling^12,18^. We and others have shown that unsaturated amino groups on the surface of the NP are capable of sequestering protons in the lysosomes leading to activation of a proton pump v-ATPase and retention of water. This so-called “proton sponge effect” might finally result in lysosomal swelling to the point of leakage of the lysosomal content and lysosomal rupture associated with apoptotic cell death^12,18,19,38,39^. The limited lysosomal leakage can be exploited in biomedical applications for lysosomal drug targeting of cancer cells^13,19,38,39^.

Consistent with previous findings^12,18,19,38,39^, PS-NH_2_ but not Si-OH induced cell death of Huh7 cancer cells (Fig. 2A). Unexpectedly, Si-NH_2_ significantly enhanced Huh7 proliferation rate (Fig. 2A). Furthermore, Huh7 cell treatment with Si-NH_2_ bearing hard protein corona (formed either by BSA or RNase) resulted in higher proliferation rate in comparison with bare Si-NH_2_ treatment (Fig. 2B–D). Importantly, cell treatment with the same concentrations used for protein corona formation of either BSA or RNase did not affect proliferation (Fig. 2B–D). Similar proliferation rate was observed in cells treated with either bare or bearing hard protein corona Si-OH NPs (Fig. 2A–D).

It is worth noting here that the adverse effects triggered by NPs were shown to be significantly ameliorated upon formation of the protein corona^37,52,53^. Consistently with these results, we found that hard protein corona had protective effects against acute toxicity induced by PS-NH_2_ NPs (Fig. 2D and Fig. S1 in Supporting Information).

### Conformational changes of adsorbed BSA and RNase proteins on functionalized nanoparticles

It has been shown that changes in protein structure and function occur as a result of their interaction with the NP surface^12^. Thus, we hypothesized that such potential changes in protein structure upon interaction with NP may be responsible for either stimulation or inhibition of Huh7 cells proliferation. Moreover, it has been showed that activity of RNase A is linked with its ability to modulate cell proliferation^54^.

**Figure 3:**
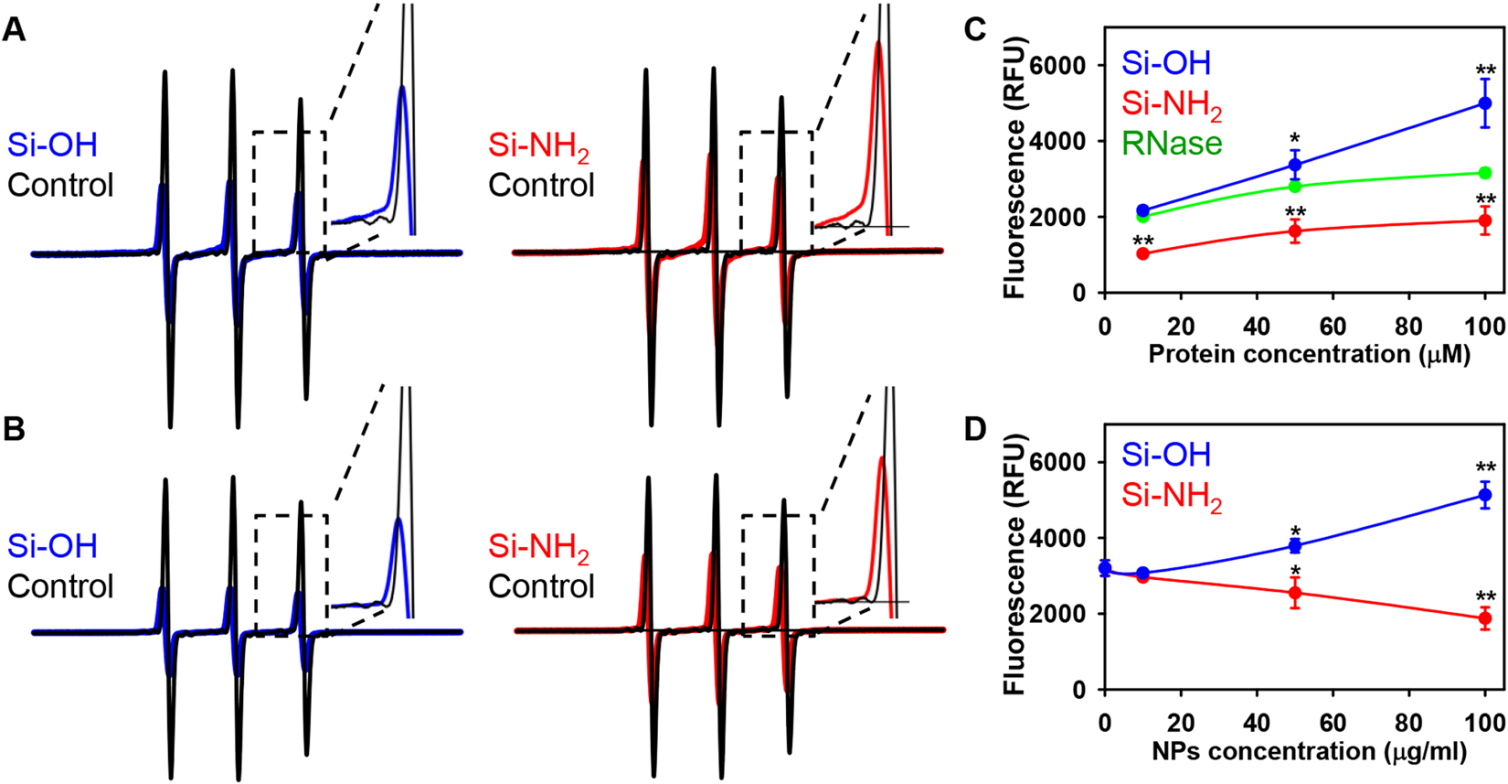
EPR spectra of (**A**) BSA and (**B**) RNase labelled with bromoacetamido-methylproxyl spin label (black traces). Labeled proteins adsorbed on silica Si-OH (blue traces) and Si NH_2_ (red traces) NPs. All spectra have been recorded under the same instrumental conditions: microwave frequency, 9.3 GHz; modulation amplitude, 1 G; modulation frequency, 100 kHz; microwave power, 1 mW; time constant, 163 ms; 30 scans; and T = 25 °C. RNase activity in presence of functionalized silica NPs as function of protein concentration (**C**) or NP concentration (**D**). (**C**) 100 µg/ml of either Si-OH or Si-NH_2_ NPs were incubated with increasing RNase concentrations in PBS pH 7.4 for 1 h. RNase activity was assessed using Ambion® RNaseAlert® Lab Test kit (Thermo Fisher Scientific), according to the manufacturer’s instruction. The kit contains a fluorescent substrate that emits a green fluorescence if it is cleaved by RNase. The data expressed as mean ± SEM, n = 3 each. *p < 0.05, **p < 0.01. (**D**) Different concentrations of either Si-OH or Si-NH_2_ NPs were incubated with RNase (100 µM) in PBS pH 7.4 for 1 h. RNAse activity was assessed as in (**C**). The data expressed as mean ± SEM, n = 3 each. *p < 0.05, **p < 0.01.

Firstly, we checked whether proteins change their conformation after adsorption to the NP surface. In order to do this, we labelled BSA and RNase with the bromoacetamidomethylproxyl spin label (SL) that reacts selectively with His residues. As a consequence, in both cases, selective spin labeling of His residues provides a tool for assessing the interaction between the NP surface and specific patches on the protein surface^55^. EPR spectra of BSA and RNase adsorbed on either Si-OH or Si-NH_2_ NPs suggest an important reduction in the mobility of the spin label of both proteins on both types of NPs (Fig. 3A,B). This means that, in both cases for both proteins, the SL-containing protein domain is directly involved in the interaction with the NP surface. Furthermore, there was a substantial shift of EPR spectra of both proteins adsorbed on either Si-OH or Si-NH_2_ NPs (Fig. 3A,B). Taking together, spin labelled BSA and RNase adsorbed on either Si-OH or Si-NH_2_ NPs show distinct spectral patterns arising from distinct local environment of the SL. In both cases, some rearrangements of the adsorbed molecules are likely to occur as well on both types of nanoparticle.

Further, we checked whether such conformational rearrangements of RNase adsorbed on the NPs could lead to changes in RNase activity. Indeed, adsorption of RNase on either Si-OH or Si-NH_2_ NPs resulted in significant activity changes as measured using specific fluorescent substrate that emits fluorescence upon cleavage by RNase (Fig. 3C,D). Moreover, RNase adsorption on Si-OH NPs significantly increased enzymatic activity (Fig. 3D). In contrast, RNase adsorbed on Si-NH_2_ NPs showed decreased enzymatic activity (Fig. 3D). This observation is not surprising since it is known that RNase changes its activity differently during adsorption onto either “positively” or “negatively” charged surfaces^56^. This happens due to the fact that RNase molecules orient differently on “positively” or “negatively” charged surfaces^56^. However, even though RNase adsorption on Si-OH and Si-NH_2_ NPs changed the enzymatic activity significantly (Fig. 3D), the changes were too low to have any noticeable impact on cellular proliferation. It has been shown that RNase enzymatic activity should change order of magnitude to affect cellular proliferation^57,58^. Furthermore, such changes in enzymatic activity are accompanied with formation of RNase A aggregates (dimers, trimers, tetramers, pentamers and hexamers)^57,58^. RNase adsorption on either Si-OH or Si-NH_2_ NPs did not increase any aggregates formation (Fig. S2 in Supporting Information). Therefore, we focused our research on other possible molecular mechanisms that could explain differential effects of Si-NH_2_ and PS-NH_2_ NPs on cellular viability.

### PS-NH_2_ but not Si-NH_2_ nanoparticles induce lysosomal permeabilization

**Figure 4:**
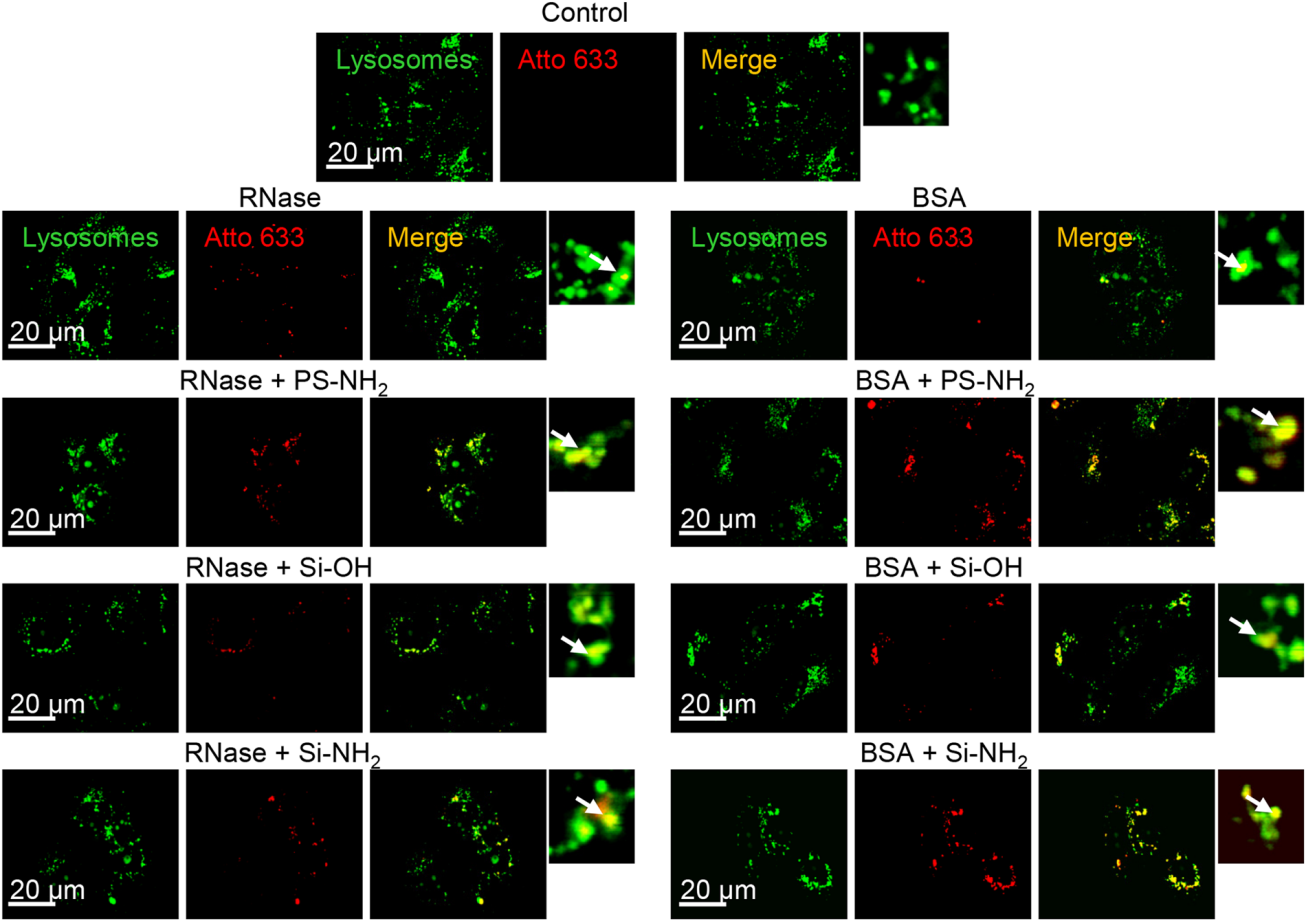
Functionalized nanoparticles bearing hard protein corona colocalize with lysosomes in Huh7 cells. Cells were incubated with BSA or RNase (both 50 µM) labelled with Atto633 (red dye) for 1 h. Additionally, cells were incubated with either Si-OH or Si-NH_2_ or PS-NH2 NPs (all 50 µg/ml) bearing either BSA or RNase as hard protein corona (both 50 µM). Acidic organelles were stained with LysoTracker probe (Invitrogen, green dye), and the cells were analyzed using confocal microscopy. Colocalization is yellow.

**Figure 5:**
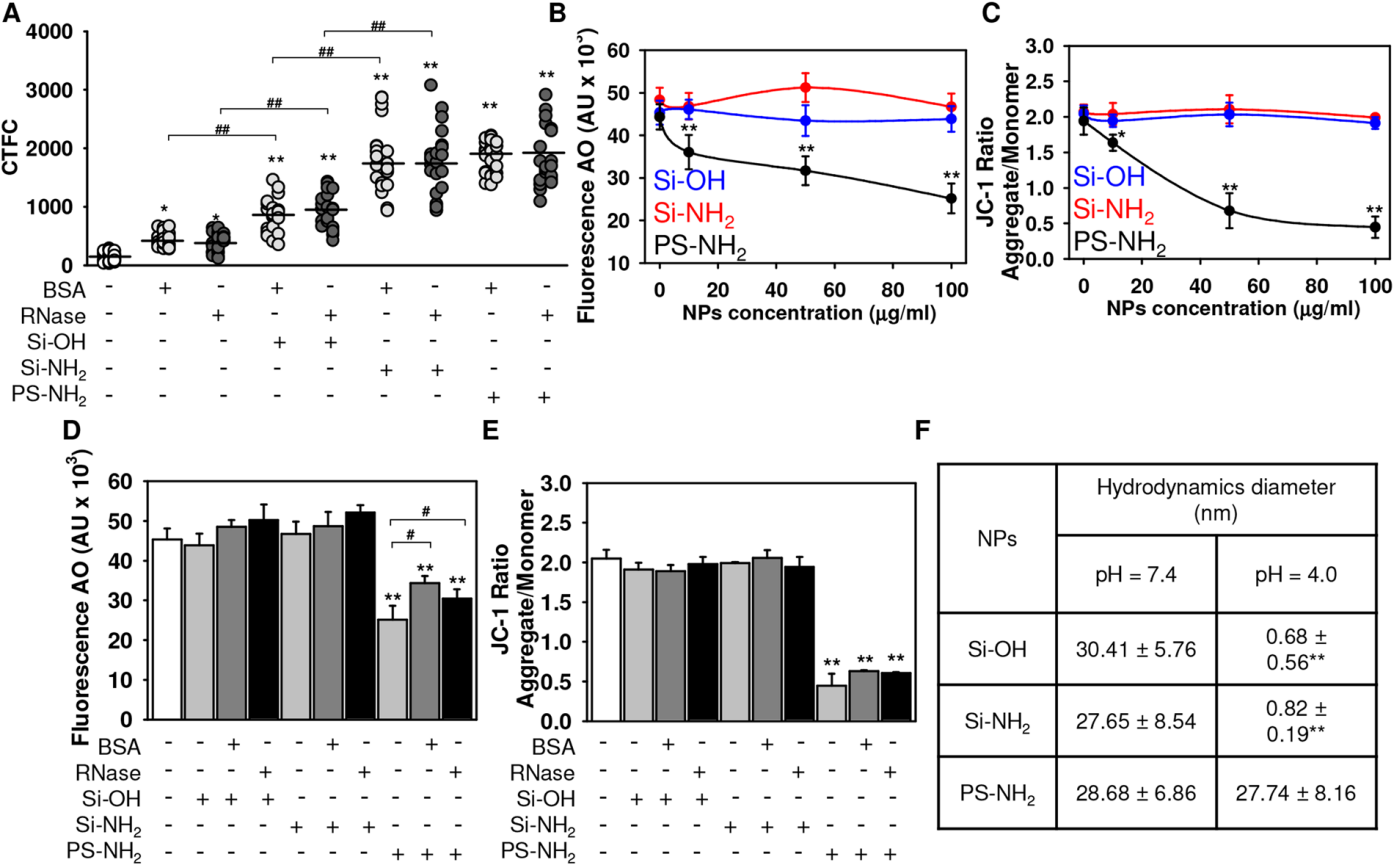
Quantification of the uptake of nanoparticles having protein corona. (**A**) Increased cell fluorescence due to accumulation of fluorescent NPs is presented as corrected total cell fluorescence (CTCF). Cells were treated as in Fig. 4. Quantifications performed using ImageJ are presented as means of n = 30 cells. *p < 0.05, **p < 0.01, ^#^ *p* < 0.05, ^##^ *p* < 0.01. (**B**) PS-NH_2_ NPs induce lysosomal leakage. Cells were treated with different NPs (0-100 µg/ml) for 4 h, stained with acridine orange (AO) and then, orange fluorescence intensity was measured using a fluorescent microplate reader (Tecan Infinite® 200 PRO). Readings were done in quadruplicates. The data expressed as mean ± SEM, n = 3 each. *p < 0.05, **p < 0.01. (**c**) PS-NH_2_ NPs induce mitochondrial dysfunction. Cells were stimulated with NPs as in (**b**) then stained with 2 µM JC-1 for 30 min and analyzed by fluorescent microplate reader (Tecan Infinite® 200 PRO). Readings were done in quadruplicates. The data expressed as mean ± SEM, n = 3 each. *p < 0.05, **p < 0.01. Comparison of lysosomal integrity (**D**) and mitochondrial potential (**E**) of Huh7 cultured with Si-OH, Si-NH2, or PS-NH2 NPs bearing BSA or RNase as hard protein corona or bare NPs. Cell were treated as in (**B**,**C**). The data expressed as mean ± SEM, n = 3 each. *p < 0.05, **p < 0.01. (**F**) Degradation of functionalized silica NPs in acidic conditions. Si-OH, Si-NH_2_ or PS-NH2 NPs (all 100 µg/ml) were incubated in either a buffer simulating extracellular conditions with neutral pH 7.4 or mimicking lysosomes with pH 4.0 for 2.5 h. After incubation hydrodynamic diameter of NPs was characterized by dynamic light scattering (DLS) using a Zetasizer Nano. The data expressed as mean ± SEM, n = 3 each. **p < 0.01.

To understand the mechanism of the inhibition of cell proliferation, we, firstly, have analyzed the subcellular localization of NPs bearing protein corona by confocal microscopy. Both proteins (BSA and RNase) as well as particles with hard protein corona (formed by either BSA or RNase) colocalized with acidic vesicular organelles which we visualized using LysoTracker, a dye targeted to compartments with low internal pH such as lysosomes (Fig. 4). Further, we analyzed uptake efficiency of both proteins and particles with hard protein corona (formed by either BSA or RNase) by Huh7 cells. Both types of proteins and NPs with hard protein corona (formed by either BSA or RNase) were taken up by the cells, although to a different extent (Fig. 5A). Huh7 internalized bare BSA or RNase to a lowest extent (Fig. 5A). Cells ingested about the same amounts of PS-NH_2_ or Si-NH_2_ bearing either BSA or RNase as hard protein corona (Fig. 5A). Interestingly, Huh7 took up significantly less Si-OH NPs (bearing either BSA or RNase as protein corona) than corresponding PS-NH_2_ or Si-NH_2_ (Fig. 5A). These data are in line with previously published research that shows that positively charged NPs may passively target tumor cells, even *in vivo* ^8,13,47^.

To address the possible lysosomal leakage induced by amino-functionalized NPs, we used the lysosomotropic dye acridine orange (AO). AO uptake in lysosomes leads to red fluorescence, which decreases when the dye is leaking from this compartment into the cytosol. After treatment with NPs, the cells were stained with AO and analyzed by fluorescence microplate reader. Treatment with PS-NH_2_, but not Si-OH or Si-NH_2_ NPs, induced a significant lysosomal permeabilization (Fig. 5B). It has been shown repeatedly that amino-functionalized NPs induce lysosomal damage due to the so-called “proton sponge” effect^12,13,18,19^. The postulated mechanism of the “proton sponge effect” involves sequestration of protons by the amines on the particle surface thereby keeping the proton pump working, which leads to the retention of one Cl^−^ ion and one water molecule for each proton that enters the lysosome. This process leads to lysosomal swelling and to osmotic rupture^12^. Finally, this process will result in leakage of lysosomal enzymes, such as cathepsin B^12,13,18,19^. When cathepsin B comes in contact with mitochondrial membranes, it causes mitochondrial dysfunction^59,60^. To investigate whether polystyrene particles can perturb mitochondrial function, we used the fluorescent dye JC-1. JC-1 is a lipophilic, cationic dye that can selectively enter into mitochondria and reversibly change color from green to red as the membrane potential increases. In healthy cells with high mitochondrial ΔmΦ, JC-1 spontaneously forms aggregates with intense red fluorescence. On the other hand, in apoptotic or unhealthy cells with low ΔmΦ, JC-1 remains in the monomeric form, which shows only green fluorescence. As expected, only PS-NH_2_ induced depolarization of the mitochondrial membrane as indicated by a decrease of the red-to-green fluorescence intensity ratio (Fig. 5C). In contrast, Si-NH_2_ and Si-OH NPs did not induce any significant changes in the mitochondrial membrane potential when compared to controls (Fig. 5C).

Interestingly, PS-NH_2_ bearing either BSA or RNase as hard protein corona showed significantly lower ability to induce lysosomal leakage (Fig. 5D). Formation of protein corona onto PS-NH_2_ NPs did not affect mitochondrial function (Fig. 5E). These data are perfectly in line with previously published studies showing that the adverse effects triggered by NPs (including cytotoxicity) are significantly mitigated in the presence of the protein corona^37,52,53,61^.

### Perturbation of mTOR activity by nanoparticles

So far biological effects obtained with PS-NH_2_ NPs were in line with previously published studies and supported “proton sponge” effect as a major cause of cytotoxicity^12,13,18,19^. However, we could not explain why Si-NH_2_ (NPs that have approximately the same charge and surface functionalization as PS-NH_2_) did not induce lysosomal leakage and subsequent cells death. Thus, we performed deep literature search for possible causes of the observed effects. Actually, PS NPs are hardly biodegradable^13,18,23,38,62,63^, whereas silicon based NPs exhibit considerable degree of biodegradability^32,34,64^. Importantly, it has been postulated that for biodegradable particles, the particle composition and degradation products might influence their biological effects^47,65,66^. These data and our results led us to a hypothesis that different biodegradability of PS-NH_2_ and Si-NH_2_ NPs could be a reason for opposite biological effects observed in our study.

Firstly, we checked stability of NPs in buffers representative for the extracellular space and lysosomal compartments. All three types of NPs showed stability in a buffer simulating extracellular conditions with neutral pH 7.4 (Fig. 5F). Incubation of PS-NH_2_ in buffer mimicking lysosomal environment with pH 4.0 did not result into particle core degradation (Fig. 5F). In contrast, Si-OH and Si-NH_2_ NPs incubated in acidic conditions showed significant particle core degradation, unlike in neutral environment (Fig. 5F).

**Figure 6:**
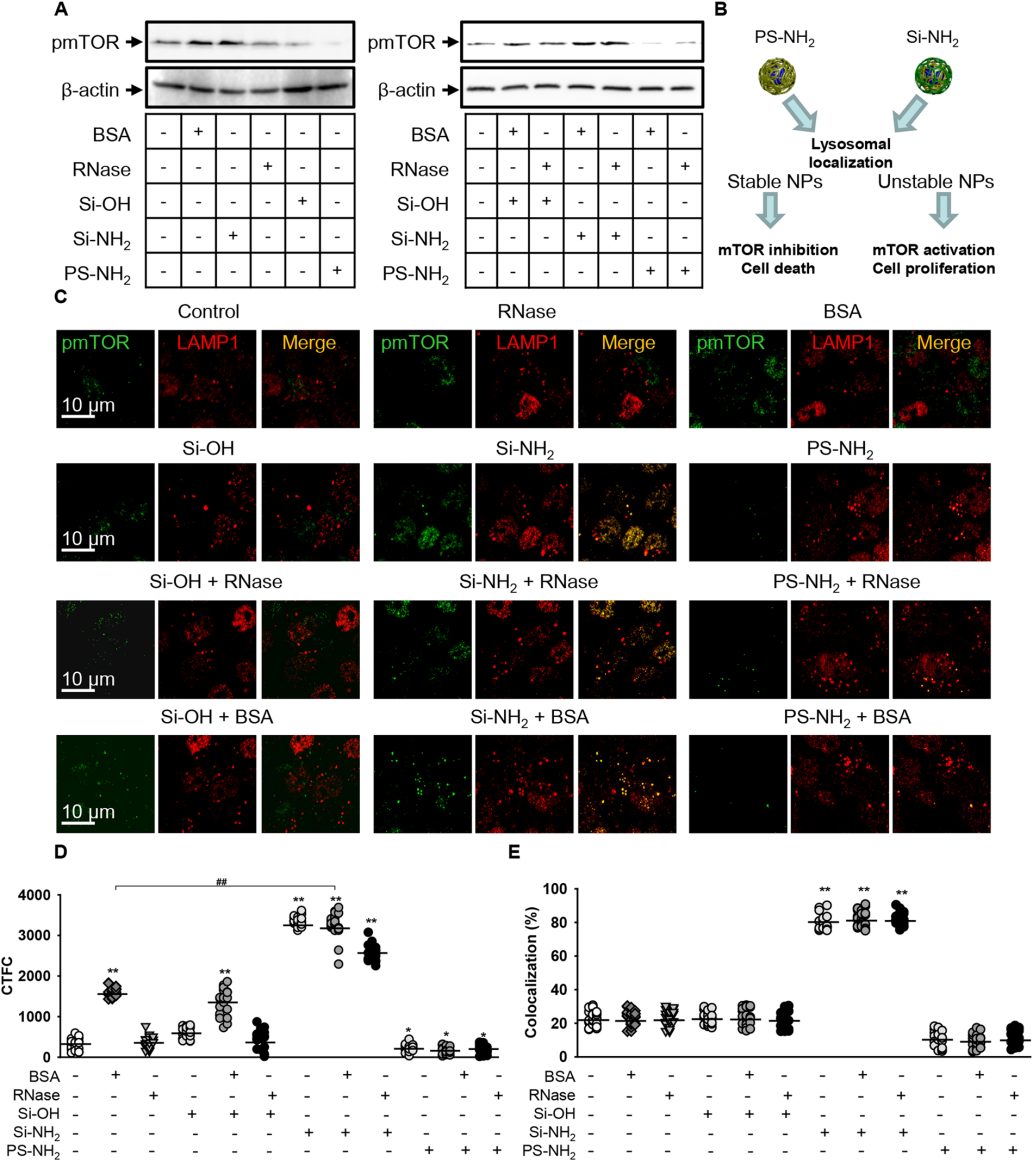
mTOR signalling in Huh7 cells exposed to different nanoparticles. (**A**) Huh7 cultured with Si-OH, Si-NH2, or PS-NH2 NPs (all 50 µg/ml) bearing BSA or RNase (both 50 µM) as hard protein corona or bare NPs for 4 h. Activation of mTOR was analyzed by Western immunoblotting (full blots are presented in Supporting Information). Actin served as a loading control. Representative blots out of 3. (**B**) Scheme illustrating the mTOR involvement in NP signalling. (**C**) Activation of mTOR on lysosomal membranes was analyzed in Huh7 stimulated as in (**A**) and analyzed by confocal microscopy using LAMP1 antibody as marker of lysosomes (red) and pmTOR (green). Colocalization is shown in yellow. Increased cell fluorescence due to mTOR phosphorylation is presented (**D**) as corrected total cell fluorescence (CTCF). Quantifications performed using ImageJ are presented as means of n = 30 cells. *p < 0.05, **p < 0.01, ^#^ *p* < 0.05, ^##^ *p* < 0.01. Colocalization analysis of pmTOR and LAMP1 from images (**C**) is presented in (**E**). Quantifications performed using ImageJ are presented as means of n = 30 cells. *p < 0.05, **p < 0.01, ^#^ *p* < 0.05, ^##^ *p* < 0.01.

Membranes of acidic organelles are important sites for activation of mTOR, a serine/threonine protein kinase that controls cell growth and cell proliferation, as well as cell motility and survival through regulation of protein synthesis and transcription^20,67^. It is well known that amino acids activate mTOR Complex 1 leading to cellular growth through increased ribosome biogenesis and elevated rates of protein synthesis, while suppressing autophagy^68,69^. Active mTOR within the mTOR complex is phosphorylated on serine 2448^70^. It has been shown that polystyrene NPs surface-functionalized with amino groups, but not those with carboxyl groups, obstruct the mTOR signaling in leukemia cells^19^. Importantly, mTOR signaling has a critical role in the pathogenesis of hepatocellular carcinoma (HCC)^26,71^. Therefore, we hypothesized that PS-NH_2_ and Si-NH_2_ NPs might differently affect this crucial signaling axis due to different stability in acidic organelles. Control Huh7 cells, indeed, exhibited activated mTOR phosphorylated on serine 2448 (Fig. 6A). Treatment with PS-NH_2_, as expected, resulted in the inhibition of mTOR phosphorylation (Fig. 6A). Differently, Si-NH_2_ NPs induced the mTOR phosphorylation (Fig. 6A). Moreover, PS-NH_2_ bearing either BSA or RNase as hard protein corona showed significant decrease in phosphorylation of the mTOR (Fig. 6A) pointing to activation of mTORC1 signaling axis. Increase in phosphorylation of the mTOR induced by addition of BSA or RNase is not surprising, since mTOR represents a nutrient sensor that is crucially important for licensing cell growth driven by oncogenic PI3K–AKT signaling and for suppressing autophagy^21,72^.

We developed further our hypothesis, that Si-NH_2_ NPs degrade in acidic organelles and this degradation is accompanied with mTOR activation. In contrast to the Si-NH_2_ NPs, PS-NH_2_ NPs were stable in lysosomes and resulted into lysosomal permeabilization leading to mTOR inhibition and subsequent cell death (Fig. 6B).

It is known that amino acids signal to the mTOR complex I growth pathway^73–75^. Specifically, stimulation of cells with positively charged amino acids (like arginine) activates mTOR signaling^73^. Furthermore, a putative lysosomal arginine sensor required for arginine to activate mTOR was recently identified^73,76^. Therefore, we thought that degradable Si-NH_2_ NPs might work as positively charged amino acids activating mTOR signaling.

First of all, confocal fluorescence microscopy of phosphorylated mTOR revealed that the majority of activated mTOR in cells treated with Si-NH_2_ was associated with lysosomal marker LAMP1, whereas in cells treated with PS-NH_2_, the pmTOR staining was weak, diffuse, and did not colocalize with lysosomes (Fig. 6C). Interestingly, BSA alone as well as Si-OH bearing BSA as hard protein corona showed significant increase in phosphorylation of the mTOR (Fig. 6D). However, this phosphorylation increase was not accompanied by colocalization with lysosomal marker LAMP1 (Fig. 6C,E). Contrarily, cells treated with Si-NH_2_ exhibited both phosphorylation increase of mTOR and colocalization with lysosomes (Fig. 6C–E). These data show that the mTOR activation in Si-NH_2_-treated cells occurs at membrane of acidic organelles, whereas these organelles in PS-NH_2_-treated cells are defective and not capable of mTOR activation.

**Figure 7:**
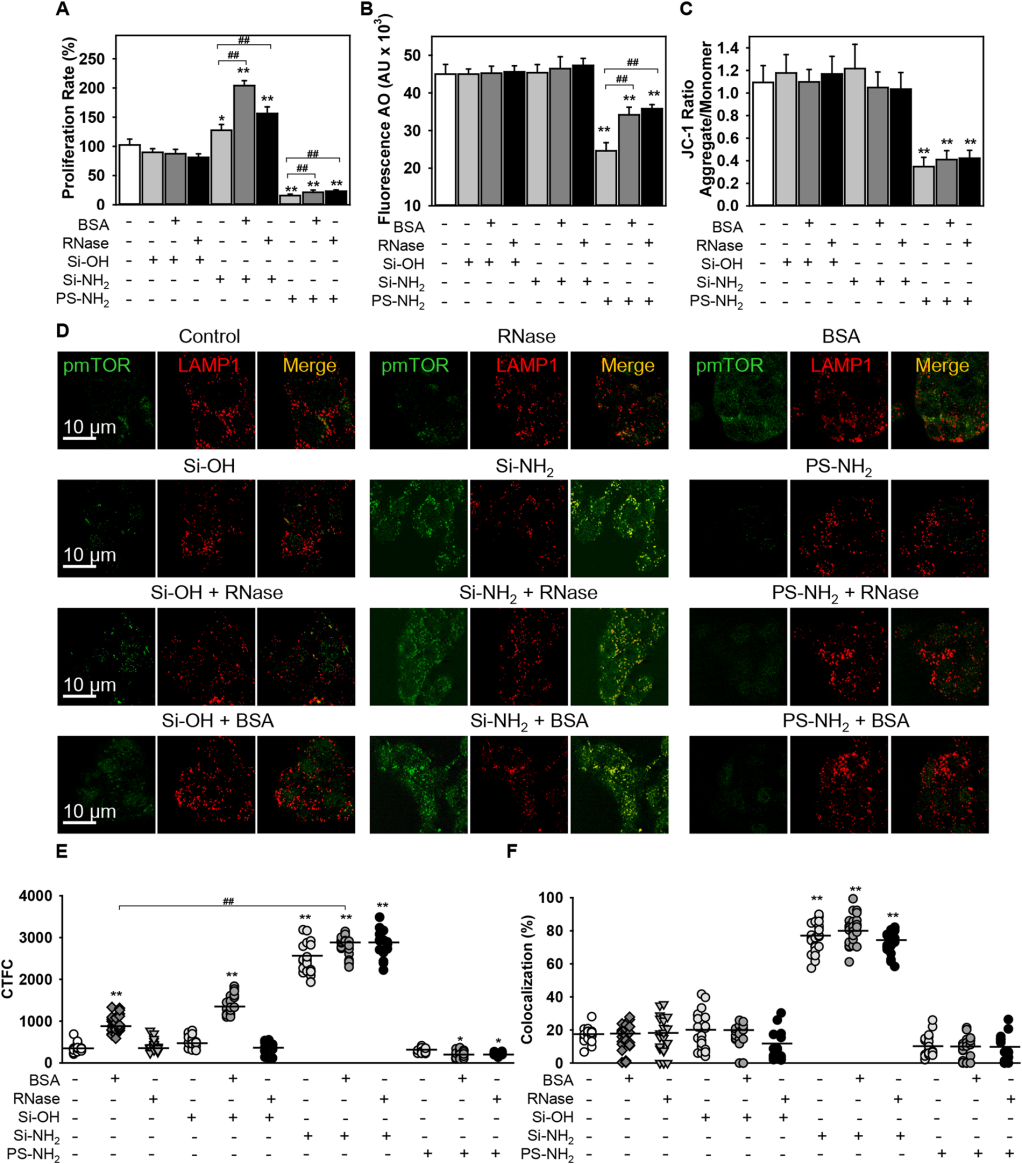
Inhibition of proliferation in HepG2 cell line by PS-NH2 nanoparticles. (**A**) Comparison of proliferative activity of HepG2 cultured with Si-OH, Si-NH_2_, or PS-NH_2_ NPs bearing BSA or RNase (100 µM both) as hard protein corona or bare NPs. Cells were cultured in medium for 24 h in the presence or absence of hydroxyl- (Si-OH), amino-functionalized (Si-NH_2_) silica, or amino-functionalized (PS-NH_2_) PS NPs. Cell viability was assessed by the WST-1 assay. The data were normalized to control values (no particle exposure) and expressed as mean ± SEM, n = 3 each. *p < 0.05, **p < 0.01, ^#^ *p* < 0.05, ^##^ *p* < 0.01. (**B**) PS-NH_2_ NPs induce lysosomal leakage. Cells were treated with different NPs (100 µg/ml) for 4 h, stained with acridine orange (AO) and then, orange fluorescence intensity was measured using a fluorescent microplate reader. The data expressed as mean ± SEM, n = 3 each. **p < 0.01. (**C**) PS-NH_2_ NPs induce mitochondrial dysfunction. Cells were stimulated with NPs as in (**B**) then stained with 2 µM JC-1 for 30 min and analyzed by fluorescent microplate reader. The data expressed as mean ± SEM, n = 3 each. **p < 0.01. (**D**) HepG2 cultured with Si-OH, Si-NH2, or PS-NH2 NPs (all 50 µg/ml) bearing BSA or RNase (both 50 µM) as hard protein corona or bare NPs for 4 h and analyzed by confocal microscopy using LAMP1 antibody as marker of lysosomes (red) and pmTOR (green). Colocalization is shown in yellow. Increased cell fluorescence due to mTOR phosphorylation is presented (**E**) as corrected total cell fluorescence (CTCF). Quantifications performed using ImageJ are presented as means of n = 30 cells. *p < 0.05, **p < 0.01, ^##^ *p* < 0.01. Colocalization analysis of pmTOR and LAMP1 from images (**D**) is presented in (**F**). Quantifications performed using ImageJ are presented as means of n = 30 cells. **p < 0.01.

To support further our findings, we re-evaluated the effects of NPs using a second liver derived cell line (HepG2). Consistent with the findings in Huh7 cells, PS-NH_2_ but not Si-OH NPs induced cell death of HepG2 cells (Fig. 7A). Moreover, Si-NH_2_ NPs significantly enhanced HepG2 proliferation rate (Fig. 7A), and HepG2 cell treatment with Si-NH_2_ bearing hard protein corona (formed either by BSA or RNase) resulted in higher proliferation rate in comparison with bare Si-NH_2_ treatment (Fig. 7A). All these findings are perfectly in line with the data obtained with Huh7 cells depicted in Fig. 2D. Additionally, treatment with PS-NH_2_, but not Si-OH or Si-NH_2_ NPs, induced a significant lysosomal permeabilization (Fig. 7B) which resulted in depolarization of the mitochondrial membrane (Fig. 7C). Further, majority of activated mTOR in HepG2 cells treated with Si-NH_2_ was associated with lysosomal marker LAMP1, whereas in cells treated with PS-NH_2_, the pmTOR staining was weak, diffuse, and did not colocalize with lysosomes (Fig. 7D). Also similarly to Huh7 treatment (Fig. 6D), BSA alone as well as Si-OH bearing BSA as a hard protein corona showed significant increase in phosphorylation of the mTOR in HepG2 (Fig. 7E). This phosphorylation increase was not accompanied by colocalization with lysosomal marker LAMP1 (Fig. 7D,F). Contrarily, HepG2 cells treated with Si-NH_2_ exhibited both phosphorylation increase of mTOR and colocalization with lysosomes (Fig. 7D–F). Thus, all data presented in Fig. 7 obtained with the second liver derived cell line HepG2 replicated the findings in Huh7.

**Figure 8:**
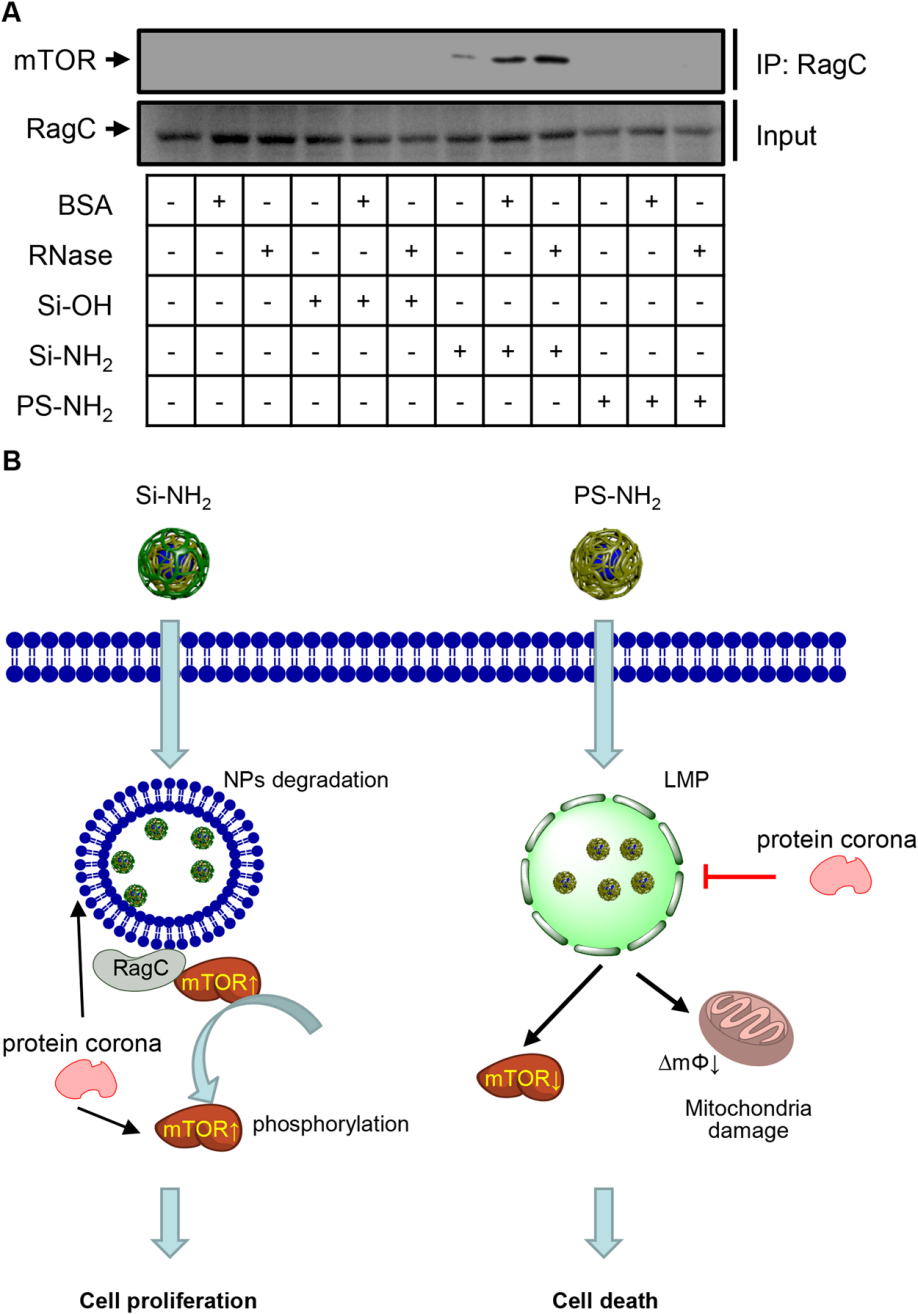
RagC complexes isolated by immunoprecipitation from Huh7 cells. (**A**) Huh7 cultured with Si-OH, Si-NH2, or PS-NH2 NPs (all 50 µg/ml) bearing BSA or RNase (both 50 µM) as hard protein corona or bare NPs for 4 h. After 4 h post NP treatment, cells were lysed with lysis buffer from immunoprecipitation kit (Abcam). RagC-mTOR complexes were co-immunoprecipitated from the precleared cell lysates with appropriate Ab as described in the manufacturer’s instruction. The resulting protein complex was eluted from the beads with Laemmli protein sample buffer for SDS-PAGE (Bio-Rad) and resolved on SDS-PAGE with specific antibody against mTOR (Cell Signaling) (full blots are presented in Supporting Information). (**B**) Scheme of district biochemical signaling activation in cells after stimulation with amino-functionalized non-biodegradable PS and biodegradable silica NPs. ∆mΦ – mitochondrial membrane potential; LMP – lysosomal membrane permeabilization.

Number of studies show that mTOR senses amino acids through the RagA–D (in particular RagC) family of GTPases^75,77,78^. Importantly, RagC makes complex with mTOR that targets the lysosomal surface and this complex formation is necessary for mTOR pathway activation^74^. Thus to check the hypothesis that Si-NH_2_ NPs might act as mTOR activator, we performed co-immunoprecipitation assay of RagC complexes. Indeed, only in cells treated with Si-NH_2_ NPs, either bare or bearing hard protein corona, endogenous RagC co-immunoprecipitated with endogenous mTOR (Fig. 8A). Taken together, these data clearly show that Si-NH_2_ NPs activate mTOR signaling whereas PS-NH_2_ leads to mTOR inhibition. Since, mTOR is regulated by the Ragulator-Rag GTPases complex, which assesses free amino acid content within the lysosomal lumen by measuring the efflux of specific amino acids across the lysosomal membrane^72^, degradable Si-NH_2_ NPs likely affect cellular functions in the same way.

## Discussion

Previously, we have analyzed the mechanisms of functionalized NP uptake by different cell lines as well as cytotoxic potential of amino-functionalized NPs^13,38^. We and others showed that unsaturated amino groups on the surface of the NP are capable of sequestering protons in the lysosomes leading to activation of a proton pump v-ATPase and retention of water. This so-called “proton sponge effect” might finally result in lysosomal swelling to the point of leakage of the lysosomal content and lysosomal rupture associated with apoptotic cell death^12,18,19,38,39^. To assess the effect of protein corona and particle functionalization on proliferation of cancer cells, we have used human liver-derived cell lines. It is worth noting here, that the liver cancer is the second most lethal cancer after pancreatic ductal adenocarcinoma in terms of 5-year survival rate^79^. Among all primary liver cancers worldwide hepatocellular carcinoma (HCC) accounts for up to 90%, and represents a major health problem^79,80^. Despite substantial advances in development of new therapies, HCC still possesses serious therapeutic challenge and targeted therapies only provide a modest benefit in terms of overall survival^80,81^.

Summarizing the data obtained in this study, we propose the following model of different NPs action on proliferation liver tumor cells (Fig. 8B). Si-NH_2_ are rapidly internalized by the studied cells with subsequent localization to lysosomes (Fig. 8B). Due to inherited instability, Si-NH_2_ NPs are rapidly degraded by lysosomal content. This degradation results in RagC-mTOR complex formation and targeting of this complex to the lysosomal surface (Fig. 8B) leading to mTOR activation. Activation of the mTOR pathway contributes to cell proliferation (Fig. 8B). Protein corona represents in this case additional source of nutrition and supports mTOR activation (Fig. 8B).

Different to Si-NH_2_ NPs, PS-NH_2_ NPs treated cells exhibit proton accumulation in lysosomes associated with lysosomal destabilization and damage of the mitochondrial membrane (Fig. 8B). Moreover, PS-NH_2_ NPs inhibit proliferation of liver derived tumor cells. At the molecular level, PS-NH_2_ NPs obstruct mTOR signaling (Fig. 8B) leading to cell death.

## Conclusions

Using non-biodegradable PS and biodegradable silica NPs as a model we have analyzed if surface functionalization and biodegradability might be used to control important cellular processes. We have found that by means of particle surface functionalization one could affect lysosomal stability and regulate essential cellular processes through control of mTOR kinase activation. Importantly, biodegradability of NPs plays a crucial role in regulation of essential cellular processes. Thus, biodegradable silica NPs having the same shape, size and surface functionalization showed opposite cellular effects in comparison with similar PS NPs. NPs surface-functionalized with amino and having different degree of biodegradability might provide a reliable tool to control the mTOR activation.

## Materials and Methods

### Materials and characterization of the NPs

Bovine serum albumin (BSA) and bovine pancreatic ribonuclease A (RNase) were purchased from Sigma-Aldrich. 3-(2-Bromoacetamido)-2,2,5,5-tetramethyl-1-pyrrolidinyloxy spin label was purchased from Santa Cruz.

Si-OH or Si-NH_2_ functionalized ~30 nm silica NPs were purchased from nanoComposix (San Diego, CA). Amino-functionalized 30 nm PS NPs were purchased from Nanocs (New York, NY). The particles were characterized by the medium particle size, polydispersity index (PDI), and zeta potential by using a Zetasizer Nano (Malvern Instruments); the particles were dispersed in PBS, pH 7.4, by sonification before each experiment.

### Fluorescence labeling

BSA and RNase were labeled with Atto 633 (Invitrogen) according to the original procedure provided by the manufacturer. In short, 1 mg ATTO 633 N-hydroxysuccinimidyl (NHS)-ester in DMSO was mixed with the 1 mg of protein in PBS buffer at pH 8.3 and incubated at room temperature for an hour. Under this conditions unprotonated ε-amino groups of protein lysine residues reacted with NHS-ester resulting in stable dye-protein conjugate. Free dye was removed using dialysis tube with a MW cutoff of 3500 Da.

### Fluorescence correlation spectroscopy (FCS)

Experiments were performed on a MicroTime 200 inverted confocal microscope (PicoQuant, Berlin, Germany) equipped with a water immersion objective (1.2 NA, 60 × ) (Olympus, Hamburg, Germany), a dichroic mirror Z473/635 RPC (Chroma, Rockingham, VT), pulsed diode laser (635 nm, LDH-P-C-635, 75 ps pulse width, 20 MHz repetition rate, PicoQuant), 50 μm pinhole, photon avalanche diode, 685/50 band pass filter (Chroma, Rockingham, VT), and TimeHarp 200 data acquisition card. Laser intensity was kept below 2 μW. The time-traces of fluorescence intensity fluctuations were recorded for 300 s and analysed using home-made routines in Origin (OriginLab, Northhampton, MA) as previously described^82^. The resulting autocorrelation curves were fitted with two-component model of 3D free diffusion:

$$G(\tau )=1+\frac{1}{N}\cdot (\frac{F}{1+\tau /{\tau }_{1}}\cdot \frac{1}{\sqrt{1+{S}^{2}\tau /{\tau }_{1}}}+\frac{1-F}{1+\tau /{\tau }_{2}}\cdot \frac{1}{\sqrt{1+{S}^{2}\tau /{\tau }_{2}}}),$$

where: *N* – mean number of fluorescent particles in the confocal volume; *S* = *r*_0_/*z*_0_, where *r*_0_ is the radius and *z*_0_ the half of the height of the point spread function; *τ*_1_ and *τ*_2_ – mean diffusion times of the first and second component, respectively; *F* – intensity fraction of the first component. The diffusion times of free proteins obtained in separate measurements were fixed for the mixtures of bound and unbound fractions. S value was determined based on the measurement of 5 nM Atto 633 aqueous solution. All measurements were performed in 8-well Lab-Tek chambers (Nalge Nunc, Rochester, NY) at 25 °C.

### Cell culture and measurement of cell viability

The human hepatocellular carcinoma cell line Huh7 obtained from Japanese Collection of Research Bioresources (JCRB) were cultured in EMEM medium (American Type Culture Collection, ATCC) supplemented with 10% FCS as recommended by the supplier. Cultures were kept in a humidified 5% CO_2_ atmosphere at 37 °C and the medium was changed twice a week. The HepG2 cell line (American Type Culture Collection, HB-8065) were grown in formulated Eagle’s Minimum Essential Medium (American Type Culture Collection) with 10% fetal bovine serum (FBS; PAA Laboratories) and 0.1% (v/v) penicillin/streptomycin (Sigma, St. Louis, MO). Cells were cultured in a humidified 5% CO_2_ atmosphere at 37 °C.

Cell viability was analyzed by WST-1 assay (Roche, Mannheim, Germany), which is based on the cleavage of tetrazolium salt WST-1 by cellular mitochondrial dehydrogenases, producing a soluble formazan salt. This conversion occurs only in viable cells, thus allowing accurate spectrophotometric quantification of the number of metabolically active cells in the culture. Cells were seeded onto 96-well plates at a density of 20 000 cells per well and treated with different NPs and proteins of indicated concentrations. 24 h after the treatment, WST-1 reagent was added to each dish and incubated for 2 h at 37 °C to form formazan. The absorbance was measured using a Tecan-Spectra ELISA plate reader (Mannedorf, Switzerland) at 450 nm. Readings were done in quadruplicates; three independent experiments were performed for each measurement.

### Spin labeling

BSA and RNase were labeled with the His-specific 3-(2-bromoacetamido-methyl)-proxyl spin label (3-(2-Bromoacetamido)-2,2,5,5-tetramethyl-1-pyrrolidinyloxy), according to the procedure described in^55,83^. Briefly, each protein (0.7 mM) was incubated in 100 mM sodium acetate buffer at pH 5.1 in the presence of a 10-fold molar excess of the spin label. The mixture was stirred for 40 h at 40 °C in the dark; it was subsequently filtered through a Vivaspin20 ultrafilter (cutoff = 3000 Da, Vivascience-Sartorius, Germany) and washed three times with PBS pH 7.4 in order to discard the excess of unreacted spin label. The labeled protein was then incubated in the presence of different concentration of Si-OH or Si-NH_2_ NPs. The suspensions were stirred in a thermostatic stirrer at 25 °C for 1 h and then filtered through a Vivaspin20 filter (cutoff = 30000 Da for RNase and cutoff = 100000 Da for BSA, Vivascience-Sartorius, Germany). The sample obtained by resuspension of the silica particles bearing the adsorbed protein in 500 μL of PBS at pH 7.4 was placed in a capillary tube for subsequent EPR measurements.

### EPR measurements

The EPR spectra were measured using a Bruker X-/Q-band E580 FT/CW ELEXSYS spectrometer. For the measurements the ER 4122 SHQE Super X High-Q cavity with TE011mode was used. The samples were placed into quartz tubes with a diameter of 2 mm. The experimental parameters were: micro-wave frequency 9.8756 GHz, microwave power 1.500 mW, modulation frequency 100 kHz, modulation amplitude 0.2 mT, and the conversion time 60 ms.

### RNase activity assay

Different concentration of Si-OH or Si-NH2 NPs were incubated with RNase in PBS pH 7.4 for 1 h. In order to discard the excess of unbound protein, the mixture was filtered through a Vivaspin20 ultrafilter (cutoff = 30000 Da, Vivascience-Sartorius, Germany) and washed three times with PBS pH 7.4. Subsequently, RNase activity was assessed using Ambion® RNaseAlert® Lab Test kit (Thermo Fisher Scientific), according to the manufacturer’s instruction. The kit contains a fluorescent substrate that emits a green fluorescence if it is cleaved by RNase, the fluorescence can be visually detected by short-wave UV illumination or measured in a fluorometer. Solutions with active RNase produce a green glow in the assay, whereas solutions without RNase activity do not fluoresce. Following staining, samples were analyzed using a fluorescence microplate reader (Tecan Infinite® 200 PRO). Readings were done in quadruplicates.

### Immunofluorescence staining

Cell were seeded into μ-Slide (Ibidi, Martinsried, Germany), then incubated for 4 h with NPs and proteins. After 4 h, cells were fixed in 4% paraformaldehyde in PBS for 15 min and stained for phospho-mTOR (1:100, Cell Signaling, catalogue no. 2971 S) and LAMP1 (1:100, Cell Signaling, catalogue no. 15665). Fixed cells were monitored on a Bio-Rad MRC-1024 laser scanning confocal microscope (Bio-Rad, Cambridge, MA).

### Analysis of nanoparticle and protein uptake

ATTO NHS-esters readily react with amino groups of proteins. Cells were treated with either BSA or RNase labelled with Atto633, or NPs with adsorbed proteins for 1 h. Si-OH, Si-NH_2_ or PS-NH_2_ NPs were incubated with Atto633-labelled BSA or RNase for 1 h in PBS pH 7.4. The suspensions were stirred in a thermostatic stirrer at 25 °C for 1 h and then filtered through a Vivaspin20 filter (cutoff = 30 000 Da for RNase and cutoff = 100 000 Da for BSA, Vivascience-Sartorius, Germany). The samples obtained by resuspension of the silica or PS particles bearing the adsorbed protein in PBS at pH 7.4 were used to treat cells. The LysoTracker probe (Invitrogen) was used to label lysosomes in cells, as described in the manufacturer’s protocol. Cells were monitored on a Bio-Rad MRC-1024 laser scanning confocal microscope (Bio-Rad, Cambridge, MA).

### Image analysis

ImageJ software (NIH) was used for image processing and fluorescent micrograph quantification. Cellular fluorescence intensity was calculated by normalizing corrected total cell fluorescence (CTCF) of the full area of interest to average a single cell fluorescence. The net average CTCF intensity of a pixel in the region of interest was calculated for each image utilizing a previously described method^4,84^. Colocalization analysis of phosphor-mTOR and LAMP1 was done using colocalization plugin in ImageJ.

### Assessment of lysosomal integrity by Acridine Orange (AO) release

Cells were labeled with 5 µg/ml AO in DMEM culture medium for 15 min at 37 °C. Following labeling, cells were cultured at 37 °C for indicated periods of time and intensity of orange fluorescence was then measured using a fluorescence microplate reader (Tecan Infinite® 200 PRO). Readings were done in quadruplicates.

### Quantification of mitochondrial membrane potential

Cells were incubated with NPs for 4 h followed by measuring of mitochondrial membrane potential (ΔmΦ). After 4 h of incubation, cells were loaded with 2 µM JC-1 (Invitrogen), a lipophilic cationic fluorescence dye with a dual emission wavelength for 30 min, in order to analyze the depolarization of the ΔmΦ. At low concentrations (due to low ΔmΦ) JC-1 is predominantly a monomer resulting in a green fluorescence with emission of 530 nm. At high concentrations (due to high ΔmΦ) the dye aggregates, yielding an orange emission of 590 nm. Thus a decrease in the aggregate fluorescent count displays a mitochondrial membrane depolarization whereas an increase exhibits a hyperpolarization. Following the staining, cells were analyzed using a fluorescence microplate reader (Tecan Infinite® 200 PRO). Readings were done in quadruplicates. Mean and standard deviation of JC-1 aggregate/monomer ratios were plotted for 3 independent experiments for each treatment.

### Cell extracts and western immunoblot analysis

Aliquots of whole cell lysates^85,86^ containing equal amounts of protein were separated by SDS-PAGE, transferred to PVDF membrane, probed with specific antibodies against phospho-mTOR (Cell Signaling, catalogue no. 2971 S) and detected as described^85,86^. Actin (Thermo Fisher Scientific) staining served as loading control.

### Co-immunoprecipitation

After 4 h post NP treatment, cells were lysed with lysis buffer from immunoprecipitation kit (Abcam, catalogue no. ab206996). RagC-mTOR complexes were co-immunoprecipitated from the precleared cell lysates with appropriate Ab as described in the manufacturer’s instruction. After pre-clearing with Protein A/G Sepharose® beads, the lysates were immunoprecipitated with anti-RagC antibody (Cell Signaling, catalogue no. 3360) for 12 hr and washed. The resulting protein complex was eluted from the beads with Laemmli protein sample buffer for SDS-PAGE (Bio-Rad) and resolved on SDS-PAGE with specific antibody against mTOR (Cell Signaling, catalogue no. 4517).

### Statistical analysis

Quantitative results are expressed as mean ± SEM or SD. The statistical significance of differences between the groups were determined using ANOVA Fisher’s LSD and Newman-Keuls tests. Differences were considered statistically significant at *p < 0.05.

Experiments utilizing multi-well microtitre plates (e.g. cell viability, RNase activity, lysosomal integrity, mitochondrial membrane potential) were conducted in accordance with guideline on randomization, spatial arrangement of samples and sampling number^87^. Readings were done in quadruplicates. Data were plotted for 3 independent experiments for each treatment.

For quantitative fluorescence microscopy analysis (uptake of nanoparticles, mTOR colocalization and phosphorylation) we used rigorously defined guidelines for accuracy and precision quantification^88,89^. The sample size determination was based on a statistical method described in^90^, which determines sample size for 95% confidence interval and 0.9 statistical power equal to 30. Therefore, n = 30 cells were used in quantification. Furthermore, to meet acceptable standards of data presentation^91^, quantitative fluorescence microscopy data were shown by displaying the full dataset as scatterplots.

## Electronic supplementary material

Supplementary Information
