# Supplementary material for: Nanoparticle core stability and surface functionalization drive the mTOR signaling pathway in hepatocellular cell lines
Source: Sci Rep. 2017 Nov 22;7:16049. doi: 10.1038/s41598-017-16447-6 (PMC5700114; doi:10.1038/s41598-017-16447-6)

Supporting Information Online

Nanoparticle core stability and surface functionalization drive the mTOR signaling pathway in hepatocellular cell lines

Mariia Lunova[1,2], Andrey Prokhorov [2], Milan Jirsa[1], Martin Hof[3], Agnieszka Olżyńska[3], Piotr Jurkiewicz[3], Šárka Kubinová[2,4], Oleg Lunov[2,*] and Alexandr Dejneka[2]

[1]Institute for Clinical & Experimental Medicine (IKEM), Prague, Czech Republic

[2]Institute of Physics of the Czech Academy of Sciences, Prague, Czech Republic

[3]J. Heyrovský Institute of Physical Chemistry AS CR, v.v.i., Dolejškova 2155/3, 182 23 Prague 8, Czech Republic

[4]Institute of Experimental Medicine AS CR, Prague, Czech Republic

[*] *Corresponding author.* Tel.: +420 266 05 2131; fax: +420 286 581 448.

E-mail address: lunov@fzu.cz (O. Lunov)

Supplementary Figures

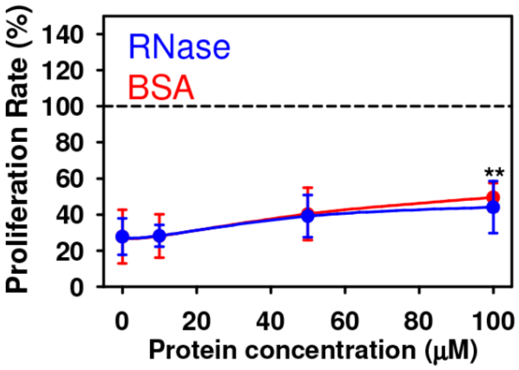

Figure S1 Analysis of cytotoxicity in Huh7 cultured with PS-NH_2 nanoparticles bearing RNase or BSA as hard protein corona. Cells were cultured in the presence or absence of PS-NH_2 nanoparticles (100 µg/ml) pre-incubated with increasing concentrations of RNase or BSA for 1 h. Cell viability was assessed by the WST-1 assay. The data were normalized to control values (no particle exposure) and expressed as mean ± SEM, n = 3 each, $^{**}p < 0.01$.

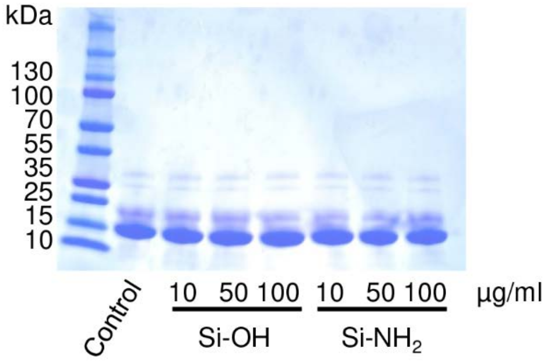

Figure S2 Analysis of nanoparticle effect on RNase dimerization. Different concentration of Si-OH or Si-NH2 nanoparticles were incubated with RNase (100 µM) in PBS pH 7.4 for 1 h. In order to discard the excess of unbound protein, the mixture was filtered through a Vivaspin20 ultrafilter (cutoff = 30000 Da, Vivascience-Sartorius, Germany) and washed three times with PBS pH 7.4. Subsequently, RNase dimers-oligomers were resolved on SDS-PAGE followed by Coomassie Brilliant Blue staining.

Figure 6A.

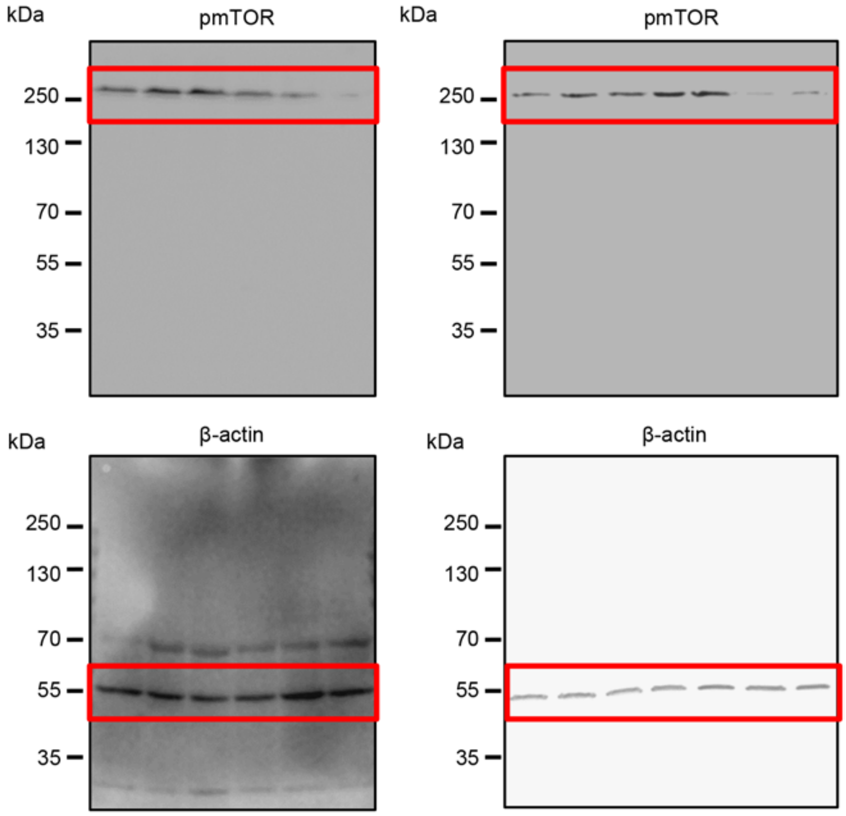

Figure 7A.

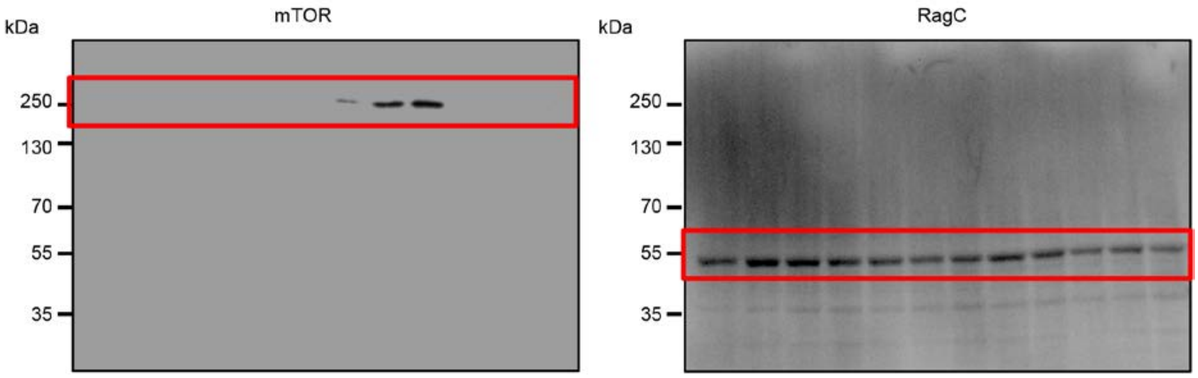

Supplement: Supplementary file 1 — Supplementary Information [file 41598_2017_16447_MOESM1_ESM.pdf]
